# Supplementary material for: Design of Rational JAK3 Inhibitors Based on the Parent Core Structure of 1,7-Dihydro-Dipyrrolo [2,3-b:3′,2′-e] Pyridine
Source: Int J Mol Sci. 2022 May 13;23(10):5437. doi: 10.3390/ijms23105437 (PMC9141313; doi:10.3390/ijms23105437)
Supplement: Supplementary file 1 [file ijms-23-05437-s001.zip › ijms-1730277-supplementary.pdf]

Supplementary material

Table S1. Variation of the number of hydrogen bonds in the composite system with time. A total of 5 frames of protein-ligand conformation were extracted at 25,000 ps intervals.

Inhibitors	Initial	MD 1ps	MD 25000ps	MD 50000ps	MD 75000ps	MD 100000ps
4	LEU905,GLU903,AGR911	LEU905,GLU903,AGR911	LEU905,GLU903	-	LEU905, GLU903	LEU905, GLU903
6	GLU903,LEU905,ARG953,ARG911	GLU903,LEU905,ARG953,ARG911,ASP967	GLU903,LEU905,ARG911	GLU903,LEU905,ARG911,ARG953	GLU903,LEU905,ARG953,GLU834	GLU903,LEU905,ARG953
8	LEU905,GLU903,LYS855,AGR953,ASP912	LEU905,GLU903,LYS855, AGR953	LEU905,GLU903,ASP967	LEU905,GLU903,ASN954	LEU905,GLU903,GLY834	LEU905,GLU903,SER835,AGR953,ASP912
10	LEU905,GLU903,ASP912, AGR953	LEU905,GLU903,ASP912, AGR953,ASP967	LEU905,GLU903	LEU905,GLU903 ,AGR953,GLY829	LEU905,GLU903, AGR953,ASP967	LEU905,GLU903,ARG911,GLY829,SER835
11	GLU903,LEU905,AGR953,AGR911	GLU903,LEU905,AGR953	GLU903,LEU905	GLU903,LEU905,AGR911,VAL836	GLU903,LEU905,, VAL836	GLU903,LEU905
17	LEU905,GLU903,AGR953,ASP912	LEU905,GLU903,AGR953	LEU905,GLU903,ASN954	LEU905,GLU903,ASN954	LEU905,GLU903,ASN954,GLY831	LEU905,GLU903,ASN954
19	LEU905,GLU903,AGR953,ASP912	LEU905,GLU903,AGR953	LEU905,GLU903,AGR953	LEU905,GLU903,AGR953	LEU905,GLU903,ARG911,ASN832	LEU905,GLU903

Supplementary material

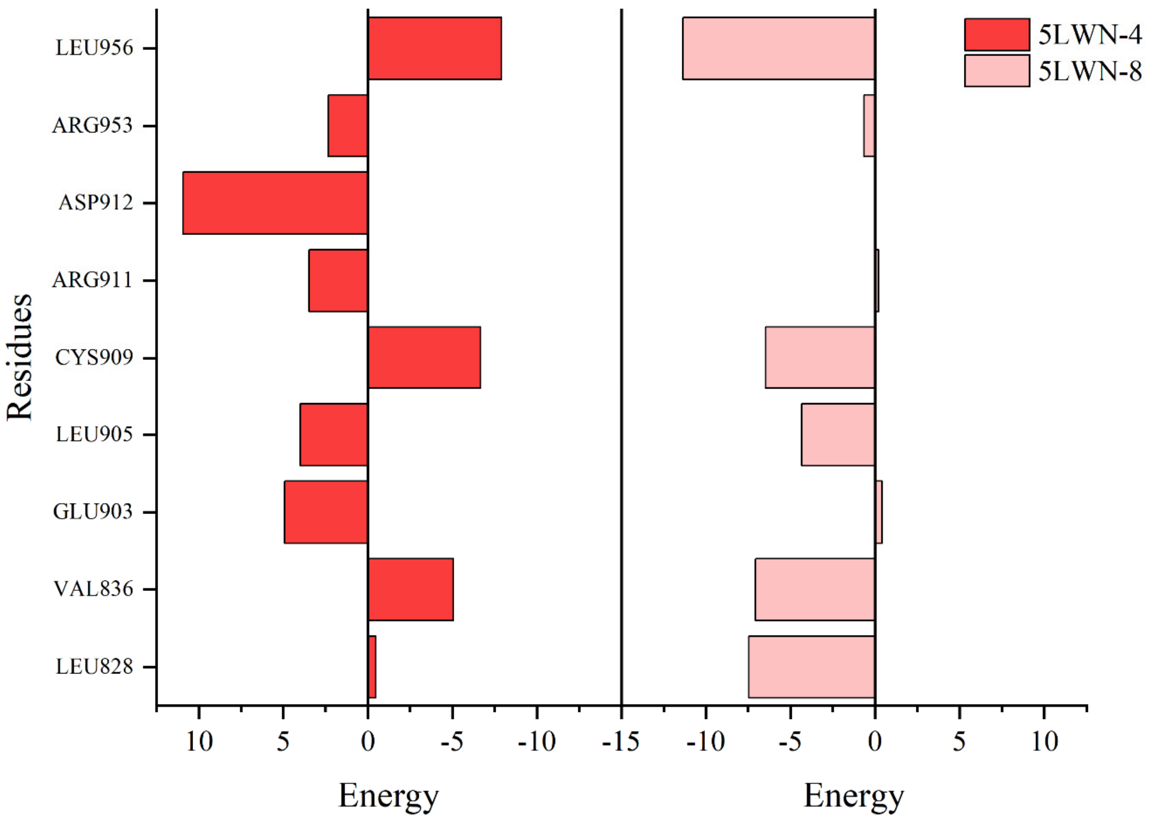

Figure S1. The decomposition of the binding energy on a per-residue basis shows only the first 9 residues that make a significant favorable contribution.

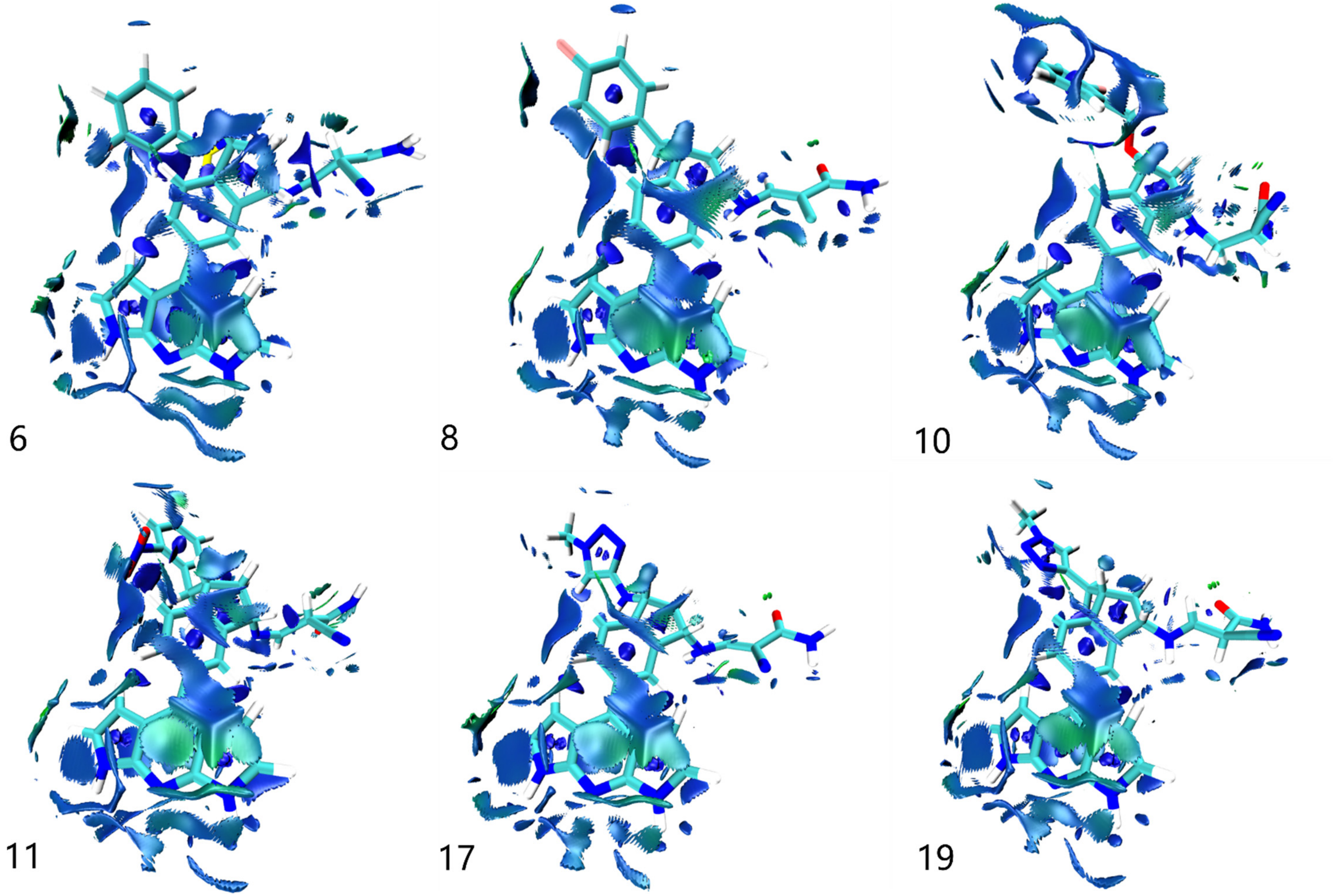

Figure S2. Thermal Fluctuation Index TFI coloring of aRDG plot. The bluer the graph, the more stable the interaction is and the lower the degree of fluctuation in the kinetic process.

Table S2. Contribution of residues around the active pocket to the binding energy.

Residues	Energy(kJ/mol)						
	4	6	8	10	11	17	19
LEU-828	-0.4799	-6.0471	-7.4616	-6.9671	-6.7221	-7.5404	-2.8286
LYS-830	1.7334	-0.8328	0.0266	-0.0217	-23.4775	-0.6668	-20.1847
VAL-836	-5.0669	-6.4899	-7.0795	-6.2699	-6.2131	-8.6077	-5.3598
ALA-853	-2.7602	-2.7194	-2.8122	1.5793	-3.1032	-2.1898	-2.7045
LYS-855	10.3545	2.9770	5.7895	-2.3547	-12.1197	7.1945	-13.4707
VAL-884	-2.0348	-1.7021	-1.6545	-1.2281	-2.2341	-1.4201	-1.1151
MET-902	-4.6243	-3.4569	-3.4590	-3.3755	-3.4786	-3.8749	-2.0766
GLU-903	4.9143	1.6816	0.4063	1.0002	17.6496	-0.6963	19.1813
LEU-905	-3.9913	-5.2908	-4.3445	-4.5726	-5.2002	-4.9015	-4.5668
CYS-909	-6.6757	-7.4228	-6.4859	-4.2445	-7.0339	-4.6843	-2.2408
ARG-911	3.4821	5.0308	0.1929	9.0244	-16.9233	1.0661	-15.3935
ASP-912	10.9343	4.7399	0.0179	0.4003	22.2801	0.2888	19.0400
ARG-953	2.3342	3.1842	-0.6573	4.2294	-14.9614	0.7846	-15.7026
LEU-956	-7.9047	-7.9906	-11.3712	-8.0315	-0.7310	-9.4455	-6.4949
ALA-966	-1.8593	-1.2590	-1.3907	-1.0103	39.9751	-1.8046	-6.4949
ASP-967	5.6042	3.9321	6.6281	11.5377	-9.1933	7.6414	59.0959

Table S3. Contribution of residues around the active pocket to the MM energy.

Residues	MM Energy (kJ/mol)	
	4	8
LEU-828	-7.0661	-14.3906
LYS-830	-3.1675	-3.2326
VAL-836	-5.7452	-7.4600
ALA-853	-3.3277	-3.8573
LYS-855	0.5354	-5.5896
VAL-884	-2.0348	-1.2219
MET-902	-4.6532	-3.5106
GLU-903	0.2640	-8.2503
LEU-905	-10.4485	-12.1427
CYS-909	-7.4074	-7.7872
ARG-911	-8.3248	0.8706
ASP-912	-3.2916	-2.1544
ARG-953	-11.7070	-3.6293
LEU-956	-8.0754	-11.3712
ALA-966	-1.8645	-1.3907
ASP-967	1.6937	6.6281
